# Bisexual Behaviors, HIV Knowledge, and Stigmatizing/Discriminatory Attitudes among Men Who Have Sex with Men

**DOI:** 10.1371/journal.pone.0130866

**Published:** 2015-06-29

**Authors:** Meizhen Liao, Mei Wang, Xingjie Shen, Pengxiang Huang, Xingguang Yang, Lianzheng Hao, Catherine Cox, Pingsheng Wu, Xiaorun Tao, Dianmin Kang, Yujiang Jia

**Affiliations:** 1 Institution for AIDS/STD Control and Prevention & Shandong Key Laboratory for Epidemic Disease Control and Prevention, Shandong CDC, Jinan, Shandong Province 250014, P. R. China; 2 Jinan Central Hospital Affiliated Shandong University, Jinan, Shandong Province 250014, P. R. China; 3 Department of Epidemiology and Biostatistics, School of Public Health, University of Maryland, College Park, MD 20742, United States of America; 4 Department of Biostatistics, Vanderbilt University, Nashville, TN 37232, United States of America; 5 Department of Health Policy, Vanderbilt University, Nashville, TN 37232, United States of America; University of Athens, Medical School, GREECE

## Abstract

**Objective:**

To assess the correlates for bisexual behaviors, HIV knowledge, and HIV/AIDS-related stigmatizing/discriminatory attitudes among men who have sex with men (MSM).

**Methods:**

A cross-sectional survey among MSM was conducted in 2011 to provide demographics, sexual behaviors, HIV knowledge, HIV/AIDS-related stigmatizing/discriminatory attitudes, and services in Jinan, Qingdao, and Yantai of Shandong Province of China.

**Results:**

Of 1230 participants, 82.8% were single, 85.7% aged <35 years, and 47.2% received college or higher education. There were 28.6% MSM who reported to be married or cohabitating or ever had sex with woman in the past 6 months (P6M). 74.5% had ≥6 HIV-related knowledge score. The average total score of stigmatizing/discriminatory attitude was 37.4±4.4(standard deviation). Bisexual behavior was independently associated with higher levels of HIV/AIDS-related stigma/discrimination(AOR = 1.1, 95% CI:1.0–1.1), older age(AOR = 1.2, 95%CI:1.1–1.2), and lower HIV-related knowledge score(AOR = 1.6, 95%CI:1.2–2.2). HIV knowledge score ≥6 was independently associated with lower levels of HIV/AIDS-related stigma/discrimination(AOR = 1.3, 95%CI:1.2–1.3), less bisexual behaviors(AOR = 0.6, 95%CI:0.5–0.9), ever received a test for HIV in the past 12 months (P12M)(AOR = 3.2, 95%CI:2.3–4.5), college or higher level education(AOR = 1.9, 95%CI:1.4–2.6), consistent condom use with men in P6M(AOR=6.9, 95%CI:4.6–10.6), recruited from internet or HIV testing sites(AOR = 11.2, 95%CI:8.0–16.1) and bars, night clubs, or tea houses(AOR = 2.5, 95%CI:1.7–4.8). Expressing higher levels of HIV/AIDS-related stigmatizing/discriminatory attitudes was independently associated with bisexual behaviors(Aβ = 0.9, 95%CI:0.4–1.4), lower HIV-related knowledge score(Aβ = 3.6, 95%CI:3.0–4.1), the number of male sex partners in the past week ≥2(Aβ = 1.4, 95%CI:1.0–1.9), unprotected male anal sex in P6M(Aβ = 1.0, 95%CI:0.5–1.6), and inversely associated with ever received HIV test(Aβ = 1.4, 95%CI:0.8–2.0) and peer education in P12M(Aβ = 1.4, 95%CI:0.9–1.9).

**Conclusion:**

HIV/AIDS-related stigmatizing/discriminatory attitudes were associated with bisexual behaviors, low HIV testing rate, lower HIV-related knowledge and risk behaviors. This study called for innovative programs that would reduce HIV/AIDS-related stigmatizing/discriminatory attitudes and bisexual behaviors and improve the uptake of prevention service among MSM.

## Introduction

In China, sexual transmission has become the major route of transmission for HIV. Men who have sex with men (MSM) have been contributing to increasing proportions of total and new infections. MSM comprise 29.4% of China’s new HIV cases according to the 2011 national estimates [1–10]. Shandong Province, situated along the east coast of China, is the hometown of Confucius and the second most populous province in China. Through the end of 2014, homosexual transmission accounted for nearly half of all reported HIV cases in Shandong [10]. It is estimated that about 2.2% of Chinese adult males have had sex with another male [11, 12]. Hence, the emerging HIV epidemic among the MSM population poses a major public health challenge in China.

The prevalence of bisexual behaviors among MSM varies according to a country’s culture and its social acceptance of homosexuality [13]. MSM behaviors are highly stigmatized in China under the strong influence of Confucianism and collectivism [12]. Such social environments may lead MSM to hide their sexual orientation by unwillingly engaging in heterosexual relationships [12, 14]. Our previous study [15] showed that, of 2996 MSM participants, 39.5% acknowledge bisexual behaviors, of which were more likely than MSM-only to be drug users and participate in other HIV-related risk behaviors. Bisexual behaviors may play a critical bridging role in spreading the HIV epidemic [16, 17].

Stigma and discrimination have been identified as major obstacles to effective responses to HIV since the beginning of the HIV/AIDS epidemic [18–20]. People who hold stigmatizing and discriminatory attitudes are less likely to have preventive behaviors, and more likely to have multiple sex partners, a commercial sex partner, and some other HIV-related high risk behaviors [19–22]. Li’s study conducted in Beijing, the capital and first-tier city in China, showed HIV/AIDS-related stigmatizing and discriminatory attitudes were inversely associated with recent HIV testing [19]. So far, no study has investigated bisexual behaviors and HIV/AIDS-related stigmatizing and discriminatory attitudes among MSM in China, which is critical information in controlling the growing epidemic. This study was conducted in three second-tier cities to further describe the associations between HIV/AIDS-related stigmatizing and discriminatory attitudes, bisexual behaviors and HIV-related knowledge among MSM in Shandong Province, China.

## Methods

### Study Participants and Settings

A cross-sectional study was conducted from April to June 2011 in the three second-tier cities of Jinan, Qingdao, and Yantai of Shandong Province. (The tiers of cities in China usually refer to key characteristics of the city, including its economic development, provincial Gross Domestic Product, advanced transportation systems and infrastructure, and historical and cultural significance. China’s first-tier cities usually refer to Beijing, Shanghai, Guangzhou, and Shenzhen.) Prior to the recruitment of the participants, we conducted in-depth interviews to collect background information and the venues to access them. Participants were recruited and interviewed by trained health professionals through multiple methods, such as community outreach, venue-based recruitment, HIV testing areas, peer referrals and Internet advertisement. Trained health professionals conducted the structured questionnaire-based interview with assistance from trained MSM peers. The enrollment criteria included being male, 18 years of age or older, having self-reported having had sex with another male in the past 12 months, and willing to complete the study. All potential participants were invited for eligibility assessments. Voluntary participation, anonymity, and confidentiality were ensured for all participants. China is still a relatively conservative country, and the traditional Chinese culture does not openly endorse MSM behaviors. Many MSM face strong social pressure to hide their identity and therefore become very apprehensive when signing the informed consent for fear of exposing their MSM identity. In order to reduce the refusal rate, verbal consent was obtained from those who refused to sign the written consent. Participants who did not agree to provide written or verbal consent were excluded in interviews. The written or verbal consent process was performed by two trained health professionals. This study and the consent procedure were approved by the Institutional Review Board of Shandong Center for Disease Control and Prevention.

### Measures

We collected data on demographics, sex and drug use behaviors, HIV knowledge, HIV testing, HIV-related prevention services, and stigma and discriminatory attitudes towards people living with HIV/AIDS (PLWHA). Bisexual behaviors were defined as participants who were married or cohabitating with a woman or who reported having had sex with a woman in the past 6 months (P6M). Eight HIV-related questions including HIV transmission modes and prevention as well as misperceptions were used to assess HIV knowledge level. Questions included “A person infected with HIV can be recognized by appearance?”; “Mosquito bites can spread HIV/AIDS?”; “Eating together can spread HIV/AIDS?” et al. All questions were weighted equally. Each correct and incorrect answer was combined into an overall score with a range of 0–8. Two groups were stratified according to whether they got a six or greater HIV-related knowledge score. Individual attitudes towards PLWHA were measured by asking participants about their agreement and disagreement (1 = yes, 2 = no) with 22 questions, details of which are described in a previous study conducted in Thailand and Zimbabwe by Genberg BL [23] and in China by Li X [19]. This scale measured 3 dimensions of HIV-related stigma and discrimination including shame, blame and social isolation. Questions included “People living with HIV/AIDS should be ashamed”; “People with AIDS should be isolated from other people”; “People who have HIV/AIDS are cursed”; “A person with HIV/AIDS should be allowed to work with other people” et al. Questions were summed to create total scale scores with a range of 22–44, where a higher score indicated a lower level of HIV/AIDS-related stigma and discrimination. Serum samples were screened for HIV-1 antibodies by enzyme-linked immunosorbent assay and confirmed as positive by Western Blot test. Syphilis screening was performed by rapid plasma reagin and confirmed by the *Treponema pallidum* particle agglutination assay.

### Statistical Analysis

We recorded the questionnaire-based data and biological testing results into the EpiData software (EpiData 6.4 for Windows). The Statistical Program for Social Sciences software (SPSS software, Version 15.0) was utilized for all analyses. Descriptive statistics were expressed as frequencies and proportions for categorical variables, and mean and standard deviations for continuous variables. Bisexual behaviors (yes/no), HIV knowledge level (≥6 or <6), and HIV/AIDS-related stigmatizing/discriminatory attitudes are the outcomes of interest. Logistic regression for bisexual behaviors and HIV knowledge level, and linear regression for HIV/AIDS-related stigmatizing/discriminatory attitudes were performed. Univariate analyses were conducted to estimate the relationship between potential risk factors and bisexual behaviors, HIV knowledge level, and HIV/AIDS-related stigmatizing/discriminatory attitudes. We further conducted stepwise backward sequence analyses to select correlates that are independently associated with these outcomes. All variables in the final multivariable models had a p-value < 0.05 and were considered significant. Adjusted odds ratios (AOR) and 95% Confidence Intervals (CIs) for having bisexual behaviors and for a higher HIV knowledge level, and adjusted effect estimates and 95% CI for HIV/AIDS-related stigmatizing/discriminatory attitudes were reported for each explanatory variable in the final models.

## Results

### Demographics

Of 1230 participants, 82.8% were single, 85.7% were less than 35 years in age, nearly half (47.2%) received college or higher education, more than two-thirds (68.7%) self-identified as homosexual, a quarter (26.3%) self-identified as bisexual, 1.4% self-identified as heterosexual, 19.4% were non-Shandong province residents, and 2.0% belonged to a non-Han ethnic group (Table 1).

**Table 1:** Scio-demographics and HIV knowledge among men who have sex with men in Shandong Province, China.

	Total		Stigma and Discrimination	Bisexual Behaviors		HIV knowledge (Score≥6)	
Variables	N	%	±SD	N	%	N	%
Total	1230		37.4±4.4	352	28.6	916	74.5
**Demographics**							
Study sites							
Jinan	400	32.5	39.0±3.8	128	32.0	367	91.8‡
Qingdao	400	32.5	33.8±3.8‡	124	31.0	164	41.0
Yantai	430	35.0	39.4±2.9	100	23.3†	385	89.5
Recruited venue							
Bars, night clubs, or tea houses	329	26.7	36.7±4.2	99	30.1	229	69.6
Bathhouses or sauna	161	13.1	33.8±3.8‡	50	31.1	64	39.8
Outdoor cruising area	98	8.0	34.1±3.9‡	29	29.6	38	38.8
Internet or HIV testing sites	642	52.5	39.2±3.6	174	27.1	585	91.1‡
Age (years)							
<25	548	44.6	37.8±4.2	85	15.5	432	78.8
25–34	505	41.1	37.4±4.4	130	25.7	374	74.1
≥35	177	14.3	36.1±4.6‡	137	77.4‡	110	62.1‡
Marital status							
Single/separated	1018	82.8	37.7±4.2	140	13.8‡	781	76.7‡
Married or cohabitating	212	17.2	35.9±4.6‡	212	100	135	63.7
Residency							
Shandong Province	991	80.6	37.4±4.4	283	28.6	749	75.6
Non-Shandong Province	239	19.4	37.6±4.2	69	28.9	167	69.9
Ethnicity group							
Han	1206	98.0	37.4±4.4	344	28.5	892	74.0
Others	24	2.0	38.8±3.5	8	33.3	24	100†
Occupation							
Student	187	15.2	38.3±4.3	12	6.4	163	87.2
Commercial service	539	43.8	37.7±4.2	160	29.7	407	75.5
Farmer	117	9.5	38.9±4.0	48	41.0‡	103	88.0
Full time employee	268	21.8	37.0±4.3	94	35.1	191	71.3
Housework and/or unemployed	119	9.7	34.6±4.3‡	38	31.9	52	43.7‡
Education							
High school or lower	649	52.8	36.9±4.5	220	33.9‡	439	67.6
College or higher	581	47.2	38.0±4.1‡	132	22.7	477	82.1‡
Duration of live in current location (years)							
≥2	815	66.3	37.4±4.4	249	30.6*	615	75.5
<2	415	33.7	37.4±4.2	103	24.8	301	72.5
HIV-related knowledge							
Score ≥6	916	74.5	38.6±3.7	245	26.7*	-	-
Score <6	314	25.5	33.8±4.0‡	107	34.1	-	-
Self-identified sexual orientation							
Homosexual	845	68.7	37.6±4.2	124	14.7	629	74.4
Heterosexual	17	1.4	34.6±4.7	8	47.1	5	29.4‡
Bisexual	323	26.3	36.9±4.6	205	63.5‡	244	75.5
Do not know	45	3.7	39.2±3.1	15	33.3	38	84.4
Being married or cohabitating/have had sex with a woman in P6M							
Homosexual	878	71.4	37.8±4.3	-	-	671	76.4‡
Bisexual	352	28.6	36.6±4.5‡	-	-	245	69.6

Total N for each subgroup may not add up to the total due to missing data; P6M: in the past 6 months

^*^: P<0.05

†: p<0.01

‡: P<0.001

### Sexual Behaviors, Prevalence Rates of HIV and Syphilis

Of all participants, 91.4% had sex with men in the P6M, 54.3% had ≥2 male sex partners in the past week, 70.8% used a condom during their last anal sex encounter, and 31.3% consistently used condoms in the P6M with male partners; 27.2% had commercial sex with men with 29.8% consistently used condoms in the P6M, 21.5% of participants had ever sold sex to a man with only 31.1% consistently used condoms in the P6M, 23.4% had sex with a female with one third (32.5%) consistently having used condoms in the P6M, only 1.1% ever used drugs, half (50.7%) had received HIV testing in the past year, three-quarters (75.2%) and 41.3% had ever received condom promotion/HIV testing, and counseling and peer education, respectively. Of all participants, 1.6% was HIV-infected and 6.8% syphilis-infected (Table 1).

### Correlates for Bisexual Behaviors

Of the 1230 participants, 28.6% were married, cohabitating or had sex with a woman in the P6M. Univariate analysis revealed that MSM who were recruited from Jinan and Qingdao, >35 years of age, married or cohabitating, had a high school or lower education, had lived in their current residential location for ≥2 years, had a score of HIV-related knowledge <6, bisexual behaviors, age of first sexual intercourse being >20 years, consistently used condoms with paid and sold male sex partners in the P6M, and HCV positive are associated with bisexual behavior. Multivariable logistic regression analysis indicated that bisexual behaviors were associated with higher level of HIV/AIDS-related stigma and discrimination (AOR = 1.1, 95% CI: 1.0–1.1), older age (AOR = 1.2, 95% CI: 1.1–1.2), a lower HIV-related knowledge score (AOR = 1.6, 95% CI: 1.2–2.2) and were recruited from Yantai city (AOR = 0.7, 95% CI: 0.5–1.0) (Tables 2 and 3).

**Table 2:** Sex and drug use behavior, HIV prevention services, and biological outcomes among men who have sex with men in Shandong Province, China.

	Total		Stigma and Discrimination	Bisexual Behaviors		HIV Knowledge (Score≥6)	
Variables	N	%	±SD	N	%	N	%
**Sexual and drug use behaviors**							
Age of first sexual intercourse (years)							
≤20	661	53.7	38.0±4.1	149	22.5	529	80.0‡
>20	569	46.3	36.7±4.6‡	203	35.7‡	387	68.0
Sex with a man in P6M							
No	105	8.6	39.4±3.2	30	28.6	98	93.3‡
Yes	1123	91.4	37.2±4.4‡	322	28.7	817	72.8
No. of male sex partners in the past week							
<2	499	45.7	38.8±3.9	133	26.7	445	89.2‡
≥2	592	54.3	35.9±4.4‡	179	30.2	345	58.3
Condom use during sex with a man in the last sexual encounter							
Yes	794	70.8	37.6±4.3	225	28.3	606	76.3‡
No	327	29.2	36.4±4.5‡	97	29.7	209	63.9
Condom use during sex with a man in P6M							
Always	351	31.3	39.1±3.4	106	30.2	324	92.3‡
Sometimes or never	770	68.7	36.4±4.5‡	216	28.1	491	63.8
Commercial sex with a man in P6M							
Yes	306	27.2	35.7±4.2‡	100	32.7	184	60.1
No	818	72.8	37.8±4.3	221	27.0	634	77.5‡
Condom use with paid male partner during the last sexual encounter							
No	84	6.8	34.3±4.4‡	23	27.4	42	50.0
Yes	222	72.5	36.2±4.0	77	34.7	142	64.0*
Condom use with paid male sex partners in the P6M							
Always	91	29.8	38.4±3.4	44	48.4‡	79	86.8‡
Sometimes or never	214	70.2	34.5±4.0‡	55	25.7	105	49.1
Sold sex to a man in P6M							
Yes	264	21.5	36.1±4.2‡	72	27.3	162	61.4
No	966	78.5	37.8±4.3	280	29.0	754	78.1‡
Condom use with sold male sex partner the last time							
No	56	21.4	34.8±4.3‡	7	12.5†	25	44.6
Yes	206	78.6	36.4±4.1	64	31.1	135	65.5†
Condom use with sold male sex partners in the P6M							
Always	82	31.1	38.8±3.2	36	43.9‡	72	87.8‡
Sometimes or never	182	68.9	34.9±4.1‡	36	19.8	90	49.5
Sex with a woman in P6M							
Yes	287	23.4	36.8±4.5†	287	100	215	74.9
No	942	76.6	37.6±4.3	64	6.8‡	700	74.3
Condom use with female partners in the last sex act							
Yes	149	52.1	37.7±4.1	-	-	122	81.9†
No	137	47.9	35.9±4.7‡	-	-	92	67.2
Condom use with female partners in the P6M							
Always	93	32.5	38.4±3.7	-	-	82	88.2‡
Sometimes or never	193	67.5	36.1±4.6‡	-	-	132	68.4
Drug use							
No	1210	98.9	37.4±4.4	344	28.4	899	74.3
Yes	13	1.1	36.9±3.8	6	46.2	10	76.9
**HIV-related prevention services in the past year**							
Condom promotion/VCT							
Yes	925	75.2	38.1±4.1	256	27.7	670	72.4
No	305	24.8	37.2±4.4†	96	31.5	246	80.7
Received peer education							
Yes	508	41.3	38.7±4.0	131	25.8	454	89.4‡
No	722	58.7	36.5±4.3‡	221	30.6	462	64.0
Had free HIV test in the past year							
Yes	624	50.7	38.5±3.9	176	28.2	550	88.1‡
No	606	49.3	36.3±4.5‡	176	29.0	366	60.4
**Biological outcome**							
HIV status							
Negative	1208	98.4	37.4±4.3	346	28.6	898	74.3
Positive	20	1.6	38.3±4.4	5	25.0	16	80.0
Syphilis status							
Negative	1144	93.2	37.5±4.4	320	28.0	858	75.0
Positive	84	6.8	36.4±4.2*	31	36.9	56	66.7
Diagnoses of sexually transmitted diseases in the past year							
No	1111	90.5	37.4±4.3	317	28.5	823	74.1
Yes	116	9.5%	37.5±4.4	34	29.3	90	77.6

Total N for each subgroup may not add up to the total due to missing data; P6M: in the past 6 months

^*^: P<0.05

†: p<0.01

‡: P<0.001

**Table 3:** Predictors for stigma and discrimination, bisexual behaviors, HIV knowledge, HIV recent testing among men who have sex with men in Shandong Province, China.

**Model 1 Predictors for stigma and discrimination**	**Mean**±**SD**	**β (95%CI)**	**Adjusted β (95%CI)**
Bisexual behaviors	36.6±4.5	1.2 (0.6–1.7)‡	0.9 (0.4–1.4)‡
HIV-related knowledge score <6	33.8±4.0	4.9 (4.4–5.4)‡	3.6 (3.0–4.1)‡
Never received a test for HIV, P12M	36.3±4.5	2.2 (1.7–2.7)‡	1.4 (0.8–2.0)‡
Never received peer education in the past year	36.5±4.3	2.3 (1.8–2.7)‡	1.4 (0.9–1.9)‡
No. of male sex partners in past week ≥2	35.9±4.4	2.9 (2.4–3.4)‡	1.4 (1.0–1.9)‡
Unprotected male anal sex in P6M	36.4±4.5	2.8 (2.2–3.3)‡	1.0 (0.5–1.6)‡
**Model 2 Predictors for bisexual behaviors**	**N (%)**	**OR (95%CI)**	**AOR (95%CI)**
Higher level of stigma and discrimination (continuous)	36.6±4.5	1.1 (1.0–1.1)‡	1.1 (1.0–1.1)†
Aged (continuous)	27.4±7.4	1.2 (1.1–1.2)‡	1.2 (1.1–1.2)‡
HIV-related knowledge score <6	107(34.1)	1.4 (1.1–1.9) *	1.6(1.2–2.2) *
Recruited from Yantai (versus Jinan)	100 (23.3)	0.6 (0.5–0.9)†	0.7 (0.5–1.0)*
**Model 3 Predictors for HIV-related knowledge (Score** ≥6)	**N (%)**	**OR (95%CI)**	**AOR (95%CI)**
Lower level of stigma and discrimination (continuous)	38.6±3.7	1.4 (1.3–1.4)‡	1.3 (1.2–1.3)‡
Bisexual behaviors	245(69.6)	0.7 (0.5–0.9) *	0.6 (0.5–0.9)†
Ever received a test for HIV, P12M	550(88.1)	4.9 (3.6–6.5)‡	3.2 (2.3–4.5)‡
College or higher level education	477(82.1)	2.2 (1.7–2.9)‡	1.9 (1.4–2.6)‡
Consistent condom use with male sex in P6M	324 (92.3)	6.8 (4.5–10.4)‡	6.9 (4.6–10.6)*
Recruited via internet or testing sites (versus outdoor cruising)	585 (91.1)	16.2 (9.9–26.4)‡	11.2(8.0–16.1)‡
Bars, night clubs, or tea houses (versus outdoor cruising area)	229(69.6)	3.6 (2.3–5.8)‡	2.5 (1.7–4.8)†

Multivariable linear regression model was performed for stigma and discrimination (Model 1); Multivariable logistic regression analysis was applied for bisexual behaviors (Model 2) and HIV knowledge (Model 3; P6M: in the past 6 months;P12M: in the past 12 months; OR: odds ratio; 95%CI: confidence interval; AOR: adjusted odds ratio

^*^: P<0.05

†: p<0.01

‡: P<0.001

### Correlates for HIV Knowledge

Of all participants, 74.5% had a ≥6 HIV-related knowledge score. Univariate analysis revealed that MSM who were recruited from Jinan, the Internet or a HIV testing site, were ≥35 years of age, single or separated, ethnicity group, college or higher education, self-identified as non-heterosexual, age of first sexual intercourse being ≤20, never had sex with a man in the P6M, had >2 male sex partners in the past week, used a condom during their last sexual encounter and consistently used condoms when having sex in the P6M, no commercial sex with a man in the P6M, and had received peer education and HIV testing in the past year are associated with higher level of HIV knowledge. Multivariable logistic regression analysis indicated that HIV knowledge was independently associated with a lower level of HIV/AIDS-related stigma and discrimination (AOR = 1.3, 95% CI: 1.2–1.3), less bisexual behaviors (AOR = 0.6, 95% CI: 0.5–0.9), having received a test for HIV in the past 12 months (AOR = 3.2, 95% CI: 2.3–4.5), a college or higher level education (AOR = 1.9, 95% CI: 1.4–2.6), consistent condom use with a male sex partner in the P6M (AOR = 6.9, 95% CI: 4.6–10.6), being recruited from the Internet or HIV testing sites (AOR = 11.2, 95% CI: 8.0–16.1) and bars, night clubs, or tea houses (AOR = 2.5, 95% CI: 1.7–4.8) (Tables 2 and 3).

### Correlates for Stigmatizing and Discriminatory Attitude

Of all participants, the total score of stigmatizing and discriminatory attitude was 37.4±4.4. Univariate analysis revealed that MSM who were recruited from Qingdao, bathhouses, sauna or outdoor cruising area, were >35 years of age, were married or cohabitating, had a lower level of education, a lower score of HIV knowledge, bisexual behaviors, age of first sexual intercourse being >20, had sex with a man in the P6M, the number of male sex partners was ≥2 in the past week, had commercial sex with a man in the P6M, no condom use during the last sexual encounter and did not consistently use condoms in the P6M, never received condom promotion, voluntary counseling, testing and peer education, did not have a HIV test in the past year and syphilis positive were also more likely to have a negative attitude towards people living with HIV/AIDS. The multivariate linear regression model indicated that stigmatizing and discriminatory attitudes were associated with bisexual behaviors (Aβ = 0.9, 95% CI: 0.4–1.4), a lower HIV-related knowledge score (Aβ = 3.6, 95% CI: 3.0–4.1), having never received a test for HIV in the past year (Aβ = 1.4, 95% CI: 0.8–2.0), having never received peer education in the past year (Aβ = 1.4, 95% CI: 0.9–1.9), the number of male sex partners in the past week being ≥2 (Aβ = 1.4, 95% CI: 1.0–1.9), and having unprotected male anal sex in the P6M (Aβ = 1.0, 95% CI: 0.5–1.6) (Tables 2 and 3).

## Discussion

This study provided the first data to assess the correlates for bisexual behaviors, HIV knowledge, and HIV/AIDS-related stigmatizing and discriminatory attitudes among MSM in three second-tier cities of Shandong Province. Multivariable logistic regression analyses showed bisexual behaviors, HIV knowledge and HIV/AIDS-related stigmatizing and discriminatory attitudes predicted each other. The findings of this study further contribute to the deeper understanding of the role of stigma and discriminatory attitudes among MSM in China. Common negative attitudes towards PLWHA, bisexual and unprotected sex among this group has created an emerging challenge in delivering prevention services to contain the rapidly growing epidemic. Studies revealed that stigma can have significant adverse effects on health and disease transmission by promoting a delay in seeking care and reluctance to follow medical advice [20, 24–26]. Confronting the rapid expansion of the HIV/AIDS epidemic among MSM in China [1, 2], this study provides more detailed evidence and calls for innovative programs that would reduce HIV-related stigmatizing and discriminatory attitudes and risks among the MSM communities in three second-tier cities of Shandong Province.

This study showed that HIV/AIDS-related stigmatizing and discriminatory attitudes were inversely associated with having received a test for HIV in the last year. This finding is consistent with Li’s report from Beijing [19]. Studies show that HIV testing and counseling can help to substantially reduce risk behaviors [27, 28]. However, only 50.7% of participants in this study had received HIV testing in the last year. This low rate of HIV testing indicates that current HIV testing and intervention programs have not yet been carried out effectively among MSM in these three second-tier cities. Given the increase in innovative technology, e.g., Internet, mobile phone, and social media usage, more innovative avenues to promote HIV testing and linkage to care could be considered and developed within the rapidly expanding HIV prevention services among this group. This study showed that bisexual behaviors were associated with a higher level of HIV/AIDS-related stigma and discrimination. This finding is not consistent with Li’s report [19]. The operational definition of bisexual behaviors might be the reason. Li’s study defined bisexual behaviors based on a self-reported sexual orientation. However, in this study, bisexual behaviors refer to the participants who were married or cohabitating with a woman, or reported having had sex with a woman in the past six months. The latter definition may be more representative than the one that was applied in Li’s study. Another reason could be that Li's study was conducted in Beijing, the capital of China and a first-tier city; which may have differences among MSM compared to that in three second-tier cities in China. The previous study [15] reported that bisexual MSM were more likely to have higher risk behaviors than MSM-only, and it highlighted the importance of bisexual behaviors as a potential epidemiologic bridge. As the home of Confucius, MSM behaviors in Shandong are stigmatized and MSM face strong social pressure under the heavy influence of Confucianism and collectivism [12, 15]. Bisexual behaviors may further reinforce the negative attitudes towards PLWHA and exacerbate high-risk behaviors, which will, conversely, result in a higher proportion of bisexual behaviors among the MSM group. Therefore, better targeted and more innovative programs are critical to reduce HIV-related stigmatizing and discriminatory attitudes. This may also aid in decreasing bisexual behaviors and improving the availability and coverage of prevention services as well as their acceptance for unique settings, such as the second-tier cities of Shandong Province, thus preventing transmission via bisexual behaviors from their high-risk male sex partners to their wives [16, 17].

This study showed that MSM with a higher HIV knowledge were more likely to express less negative attitudes. This finding is consistent with Dias’ report [29]. Those MSM who have a higher education level will have more access to HIV-related knowledge and health information, and will have a lower perceived stigma and discriminatory social pressure. This study also found that peer education has a similar effect. Peer education could alleviate social pressure, persuade their peers to seek HIV testing and counseling, and provide a link to care and medical treatment [20, 27, 30]. The findings of this study suggest HIV-related knowledge promotion, expanding HIV testing, and peer education could be integral parts of the conventional HIV/AIDS services to reduce the stigmatization and discrimination among this group. However, this study found that high risk behaviors widely exist; 54.3% of the participants had ≥2 male sex partners in the past week, and only 31.3% consistently used condoms in the past six months with male partners. This finding suggests that greater efforts should be focused on enhancing individuals’ motivation to change their behavioral patterns and teaching behavioral skills to reduce risky behaviors. The findings also suggest HIV-related knowledge promotion strategies should vary from venue to venue.

This study highlights the importance of policy considerations for stigma and discrimination, and its related sexual risks among MSM. In spite of considerate planning, implementation and quality control, this study has its limitations. The data relying on retrospective self-reporting may be subject to recall bias. Sensitivity of sex and drug related questions and expressing stigmatizing and discriminatory attitudes towards PLWHA could lead to reporting bias and social desirability bias. In addition, the non-response information was not collected.

Despite these limitations, this study provides important information for further research and suggests that MSM who experience stigmatizing and discriminatory attitudes towards PLWHA may be at a higher risk for an increased number of sex partners, being bisexual, having a lower level of HIV knowledge, a smaller proportion of having received HIV testing and peer education, and having unprotected sex. The findings of this study call for innovative programs that would reduce HIV/AIDS-related stigmatizing and discriminatory attitudes and bisexual risk behaviors and improve the uptake of prevention services among MSM in the three second-tier cities Jinan, Qingdao, and Yantai of Shandong Province.
